# *De novo* sequencing, assembly, and characterization of *Asparagus racemosus* transcriptome and analysis of expression profile of genes involved in the flavonoid biosynthesis pathway

**DOI:** 10.3389/fgene.2023.1236517

**Published:** 2023-09-07

**Authors:** Chanchal Malik, Sudhanshu Dwivedi, Tilahun Rabuma, Ravinder Kumar, Nitesh Singh, Anil Kumar, Rajesh Yogi, Vinod Chhokar

**Affiliations:** ^1^ Department of Bio and Nano Technology, Guru Jambheshwar University of Science and Technology, Hisar, Haryana, India; ^2^ Department of Biotechnology, College of Natural and Computational Science, Wolkite University, Wolkite, Ethiopia; ^3^ Faculty of Agricultural Sciences, Shree Guru Gobind Singh Tricentenary University, Gurugram, Haryana, India; ^4^ UIBT-Biotechnology, Chandigarh University, Mohali, Punjab, India

**Keywords:** *Asparagus racemosus*, gene ontology, secondary metabolism, real time PCR, gene expression

## Abstract

*Asparagus racemosus* is known for its diverse content of secondary metabolites, i.e., saponins, alkaloids, and a wide range of flavonoids. Flavonoids, including phenols and polyphenols, have a significant role in plant physiology and are synthesized in several tissues. Despite the diverse role of flavonoids, genetic information is limited for flavonoid biosynthesis pathways in *A. racemosus*. The current study explores full-scale functional genomics information of *A. racemosus* by *de novo* transcriptome sequencing using Illumina paired-end sequencing technology to elucidate the genes involved in flavonoid biosynthesis pathways. The *de novo* assembly of high-quality paired-end reads resulted in ∼2.3 million high-quality reads with a pooled transcript of 45,647 comprising ∼76 Mb transcriptome with a mean length (bp) of 1,674 and N50 of 1,868bp. Furthermore, the coding sequence (CDS) prediction analysis from 45,647 pooled transcripts resulted in 45,444 CDS with a total length and mean length of 76,398,686 and 1,674, respectively. The Gene Ontology (GO) analysis resulted in a high number of CDSs assigned to 25,342 GO terms, which grouped the predicted CDS into three main domains, i.e., Biological Process (19,550), Molecular Function (19,873), and Cellular Component (14,577). The Kyoto Encyclopedia of Genes and Genomes (KEGG) pathway database was used to categorize 6,353 CDS into 25 distinct biological pathway categories, in which the majority of mapped CDS were shown to be related to translation (645), followed by signal transduction (532), carbohydrate metabolism (524), folding, sorting, and degradation (522). Among these, only ∼64 and 14 CDSs were found to be involved in the phenylpropanoid and flavonoid biosynthesis pathways, respectively. Quantitative Real-time PCR was used to check the expression profile of fourteen potential flavonoid biosynthesis pathway genes. The qRT-PCR analysis result matches the transcriptome sequence data validating the Illumina sequence results. Moreover, a large number of genes associated with the flavonoids biosynthesis pathway were found to be upregulated under the induction of methyl jasmonate. The present-day study on transcriptome sequence data of *A. racemosus* can be utilized for characterizing genes involved in flavonoid biosynthesis pathways and for functional genomics analysis in *A. racemosus* using the reverse genetics approach (CRISPR/Cas9 technology).

## 1 Introduction

*Asparagus racemosus*, commonly called Shatavari, belongs to the family of Liliaceae, found in low altitudes throughout India (Rani et al., 2022). It is an extensively scandent spinous, branched under-shrub. Leaves are reduced to small, and roots are numerous and fusiform succulent, and it looms in clusters from the basal end of the stem and has a tuberous shape with a diameter ranging from 0.5 to 1.5 cm. (Bopana and Saxena, 2008). Different parts of the plant exhibit different therapeutic and pharmacological properties, but roots are the most widely used as medicine and health tonic. Roots are rich in various steroids, flavonoids, alkaloids, polyphenols, vitamins, and shatavarins and are used as medicine for different ailments, such as weakness, infertility, libido, menstrual irregularity, dyspepsia, hepatitis, allergies (Prakash and Singh, 2006; Chawla et al., 2011; Kumar et al., 2011), and galactagogue, etc. (Gupta and Shaw, 2011). The dried root of the plant is used as a drug treating ulcers and has been identified to control AIDS (Alok et al., 2013).

Moreover, it is commonly used in traditional Ayurveda preparations, especially in female and rejuvenating tonics (Sharma and Bhatnagar, 2010). The complexity of biological functions arises from intricate interactions among multiple components, including the genome, gene products, and metabolites. To comprehend the molecular underpinnings of these intricate interactions, functional genomics approaches, which utilize NGS data, are promising in discovering the molecular mechanisms that drive complex biological processes involving interactions between gene products, metabolites, and the genome. With the advent of next-generation sequencing technology, genomics research has revolutionized, allowing for the efficient, cost-effective sequencing of genes. This has resulted in the identification of new genes associated with metabolic pathways, especially in non-model plants like eucalyptus (Mizrachi et al., 2010), *American ginseng* (Sun et al., 2010), rubber tree (Liu et al., 2015), *Aloe vera* (Choudhri et al., 2018) and many others that lack a reference genome.

**FIGURE 1:**
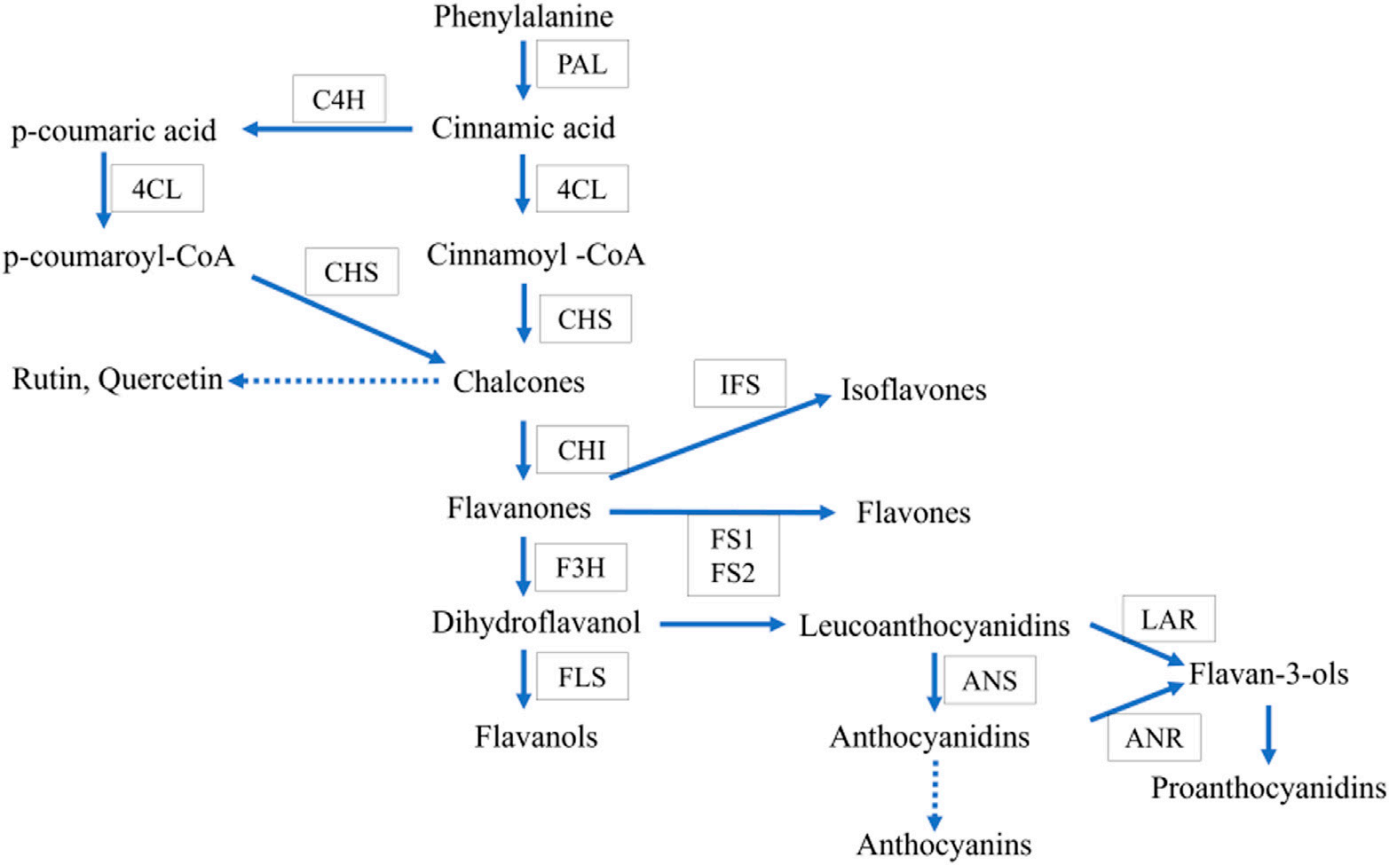
Proposed common flavonoid biosynthesis. The enzymes catalyzing each step are in boxes Phenylalanine ammonia-lyase (PAL), Chalcone synthase (CHS), cinnamate-4- hydroxylase (C4H), chalcone isomerase (CHI), flavone synthase (FS), flavonoid-3- hydroxylase (F3H), Flavonol synthase (FLS), anthocyanidin synthase (ANS), isoflavon synthase (IFS).

*Asparagus racemosus* contains several secondary metabolites such as flavonoids, alkaloids, and saponins (Hayes et al., 2008; Hossain et al., 2012; Kumar et al., 2016), thereby increasing pharmaceutics’ attention due to its multiple applications. Secondary metabolites such as quercetin, rutin, and kaempferol are the mainly reported flavonoid components in *A. racemosus*. They have numerous therapeutic activities, including anti oxidative action (Taepongsorat and Rattana, 2018), anti-inflammatory, antitumor effects (Mitra et al., 2012), and antimicrobial activities (Aggarwal et al., 2013). Flavonoids, a class of phenylpropanoids widely distributed in the plant kingdom, comprise one of the major secondary metabolite groups with a C6-C3-C6 general structural backbone. The biosynthesis of flavonoid backbone has been extensively reported in natural product chemistry and molecular biology (Ververidis et al., 2007). Irrespective of their structural variations, the origin of all flavonoid compounds can be traced back to the phenylpropanoid pathway, which converts phenylalanine into 4-coumaroyl-CoA. This is the crucial step in the flavonoid biosynthesis pathway. Chalcone synthase (CHS) is the first enzyme involved in condensation, and chalcone is formed after subsequent intramolecular cyclization of one p-coumaroyl-CoA with malonyl-CoA molecules (Figure 1). Further, chalcone isomerase (CHI) catalyzes the isomerization of chalcone into flavanone. After that, flavone synthase (FS) converts flavanone into flavone. Dihydroflavonol is formed by the 3-hydroxylation of flavanone by the Flavanone 3-hydroxylase enzyme (F3H) (Ferrer et al., 2008). However, various products, such as anthocyanins, rutin, quercetin, flavonols, *etc.*, require many steps, and sequential modifications among them are acylation, methylation, and glycosylation to build the end product. Most of these steps remain to be elucidated in plants in general and *A. racemosus* in particular.

Despite the importance of flavonoids, the genomic information related to their biosynthesis in *A. racemosus* is poorly understood. Therefore, the present study performed a *de novo* transcriptome sequencing and data assembling analysis of *A. racemosus* to elucidate the genes involved in flavonoid biosynthesis pathways and validate the transcriptomic sequencing data using qRT-PCR. Transcriptome sequencing and analysis of *A. racemosus* can pave the way for a wide array of basic and applied research. Sequencing the transcriptome of this plant will provide a comprehensive catalog of the expressed genes and transcripts, providing researchers with an extensive pool of genomic resources. This information can be used to study the molecular and biochemical processes that underlie the various physiological functions of *A. racemosus*. The application of transcriptome sequencing and analysis in *A. racemosus* research can offer a wealth of genomic resources and insights that can be utilized in various fields of study, including agriculture, medicine, and biotechnology.

## 2 Methods

### 2.1 Sample preparation and total RNA isolation

Two-year-old plantlets (*A. racemosus*) regenerated via clonal propagation (Kumar Kar and Sen, 1985) were obtained from the herbal garden of Chaudhary Charan Singh Haryana Agricultural University, Hisar, Haryana, India. Plants were cultivated in a pot that contained a mixture of sterilized soil, sand, and compost in a 2:1:1 ratio under greenhouse conditions at 24°C–28°C and a 16-h light/8-h night cycle. Fresh leaves and roots were harvested from a healthy plant, splashed frozen in liquid nitrogen, and stored at −80°C for further use. The total RNA was extracted from the root and leaves tissues using TRIzol^®^ Reagent (Thermo Fisher Scientific) method. To ensure the presence of the 28S and 18S bands, the total RNA’s quality was assessed on a 1% denatured agarose gel at 100 V for 30 min (Supplementary Figure S1). Further, the total RNA quality and quantity were analyzed using a Qubit fluorometer. Finally, the quality of RNA was evaluated using Bioanalyzer Agilent DNA HS Chip (Thermo fisher scientific).

### 2.2 Library preparation

The pair-end cDNA sequencing library was prepared by pooling 4 µg total RNA of root and leaf using TrueSeq^®^ Stranded mRNA sample preparation kit (Illumina) as per the manufacturer’s instruction. The total RNA isolated from the root and leaf was pooled in an equimolar ratio. Poly- T-attached magnetic beads were utilized to enrich mRNA from the total RNA, which was then enzymatically fragmented and converted into the first-strand cDNA. Subsequently, a second-strand mix was employed to facilitate RNA-dependent synthesis for synthesizing the second strand cDNA from the first cDNA strands. The double-stranded cDNA was purified using AMPure XP beads (Agencourt Biosciences). In the double-strand cDNAs, end repair, A-tailing, and adapter ligation were performed and finally ended with 18 cycles of PCR amplification of the adaptor-ligated library. Following the manufacturer’s instructions, the Tape Station 4200 (Agilent Technologies) and the High Sensitivity (HS) D5000 Screen Tape assay kit was utilized to analyze the PCR amplified library.

### 2.3 Illumina RNA sequencing and data trimming

For cluster generation and sequencing cDNA pair-end library prepared from the pooled sample was loaded onto Next Seq500. The library sequencing and raw data generation for both samples were performed by Xcelris Genomics, using the Illumina Hi-Seq platform with 2 × 150 bp chemistry. The raw sequencing data produced underwent filtration using Trimmomatic v0.30, which involved applying several parameters. These parameters included cutting the read once the average quality within the window falls below a threshold of 25, cutting the bases off at the beginning of the read if they are below a quality threshold of 25, cutting the bases off at the end of the read if they are below a quality threshold of 25, and discarding any reads that are below a length of 50 bp.

### 2.4 *De novo* transcriptome assembly, validation, and CDS prediction

**FIGURE 2:**
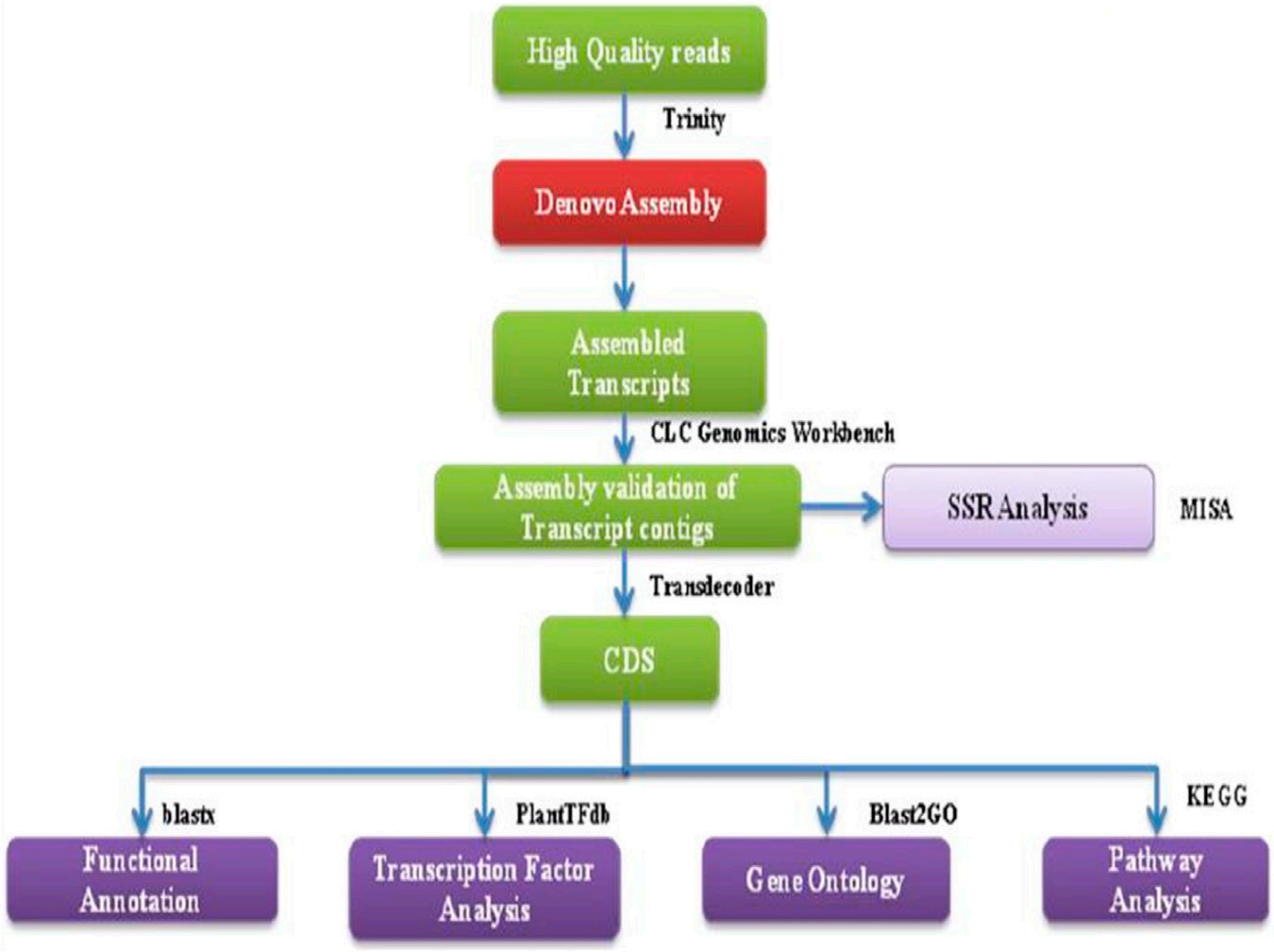
A flowchart showing the bioinformatics workflow of *de novo* transcriptome sequencing strategy and analysis for NGS-Asparagus leaf and root sample.

Following the removal of low-quality reads, the remaining high-quality reads were combined and assembled into transcripts using RNA-Seq Assembler Trinity, utilizing a Kmer 25. The validation of transcript assembly was conducted using the CLC Genomics workbench, as depicted in Figure 2. To eliminate isoforms produced during assembly and obtain unigenes that could no longer be extended, non-redundant transcripts were further clustered using CDHIT-EST-454 with 95% identity and query coverage. TransDecoder-v5.3.0 was used to predict coding sequences from unigenes, using specific criteria such as the presence of an open reading frame (ORF) with a minimum length and a log-likelihood score >0.

The coding score was most significant when the ORF was scored in the first reading frame compared to the other five frames. Subsequently, downstream analysis was conducted using CDS with 80% coverage and 3X read depth.

### 2.5 Gene ontology analysis

To annotate the predicted CDS, a search was conducted against NCBI (http://www.ncbi.nlm.nih.gov) using the Basic Local Alignment Search Tool (BLASTx) with a significance threshold cut-off of E-value ≤1e-05. Gene ontology prediction was carried out using the Blast2GO (v 2.7.0) program. The predicted CDS were further categorized into three domains representing gene product properties, namely: Biological Process (BP), Molecular Function (MF), and Cellular Component (CC), using gene ontology mapping analysis.

### 2.6 Functional annotation of KEGG pathways

To identify the predicted CDS’s possible involvement, the CDS was mapped into the reference canonical pathways Kyoto Encyclopedia of Genes and Genomes (KEGG) database (Kanehisa et al., 2004). The potential involvement of predicted CDS was categorized into 25 KEGG pathways under five significant categories, i.e., metabolism, genetic information processing, environmental information processing, cellular processing, and organismal system. The information obtained upon the KEGG pathways analysis included KEGG Orthology (KO) assignments, their corresponding enzyme commission (EC) number, and the prediction of the metabolic pathways using the KEGG automated annotation server KASS (Moriya et al., 2007).

### 2.7 Transcription factor analysis

Transcription factor identification was performed using P2TF (Predicted Prokaryotic Transcription Factors), which is an integrated and comprehensive database relating to transcription factor proteins. All the predicted CDS were searched against the Plant transcription factor database (PlantTFdb) using BLASTX to obtain the transcription factor from pooled leaf and root CDS. The transcription factors were identified within the coding regions.

### 2.8 SSR identification

The observed unigenes were assembled, and SSRs determined using the MIcroSAtellite Identification tool (MISA, http://pgrc.inpk-gatersleben.de/misa/). A minimum unit size cut-off of 6 was used to report a dinucleotide repeat; for a trinucleotide repeat size cut-off was 4, and for SSRs of sizes 4, 5, and 6 repeats, the cut-off size was 3. A maximum distance of 100 nucleotides was allowed between the two SSRs.

### 2.9 Methyl jasmonate treatment

The plantlets of control and methyl jasmonate-treated plants were grown in three biological replications. Methyl jasmonate treatment was performed with the foliar spray containing an aqueous solution of 250 µM MeJA and 0.1% Triton X-100 (Ku and Juvik, 2013).

### 2.10 Gene validation analysis using qRT-PCR

For the validation of the transcriptomic sequencing data by real-time quantitative PCR (qRT-PCR), fourteen identified genes involved in the flavonoid biosynthesis pathways were selected from *A. racemosus* transcriptome sequencing data, and primers were designed for the fourteen candidate genes, and housekeeping genes (GAPDH) using Primer Express software (version 3.0.1) (Table 1). The total RNA was isolated using the CIA-PCIA method (Choudhri et al., 2018) from the root and leaf samples and plants externally treated with methyl jasmonate (250 µM). According to the manufacturer’s protocol, the isolated RNA (1 μg) was converted to cDNA using the PrimeScript first strand cDNA Synthesis Kit. The qRT-PCR quantification was performed using the Step One Real-Time PCR Instrument (Applied Biosystems) and Takara TB Green™ Premix Ex Taq™ II (Tli RNaseH Plus). The PCR was conducted in a 20 μL volume containing 4 μL diluted cDNA, 250 nM forward primer, 250 nM reverse primer, and 10 μL TB Green Premix Ex Taq II (Tli RNaseH Plus) (2X) (Takara) using the following conditions: 95°C for 3 min, 40 cycles of 95°C for 15 s, 58°C for 15 s, 72°C for 20 s and final extension at 72°C for 5 min. Three technical replications were used for the qRT-PCR reaction of each biological sample. The housekeeping gene GAPDH was utilized as a reference gene in qRT-PCR gene validation analysis for data normalization (Jian et al., 2008). The relative expression level was determined using the 2^−ΔΔCT^ method (CT Target- CT GAPDH) (Schmittgen and Livak, 2008) based on three technical replicates of each sample.

**TABLE 1:** Selected genes involved in biosynthesis of flavonoid.

Sr No.	Gene name	EC no.	Unique sequence
1	Trans cinnamte-4-monooxygenase	1.14.13.11	cds_2444
2	Anthocyanidin-3-o- glycosyl transferase	2.4.1.115	cds_12314
3	Anthocyanidin glycosyltransferase	2.4.1.115	cds_12317
4	Shikimate hydroxyl cinnamoyl transferase	2.3.1.133	cds_14257
5	Shikimate hydroxyl cinnamoyl transferase	2.3.1.133	cds_14258
6	Isoflavanone -2- hydroxylase	1.14.13.52	cds_14383
7	Flavanol synthase	1.14.11.23	cds_14436
8	Flavanol synthase	1.14.11.23	cds_14711
9	Chalcone synthase	2.3.1.74	cds_15823
10	Caffeiol CoA methyltransferase	2.1.1.104	cds_16000
11	Naringenine-3- dioxygenase	1.14.11.9	cds_16714
12	Qumoroyl quinate monooxygenase	1.14.13.36	cds_17418
13	Flavonol monooxygenase	1.14.13.21	cds_22665
14	Flavonol glycosyltransferase	2.4.1.91	cds_34397

## 3 Results

### 3.1 *De novo* transcriptome assembly and validation

**FIGURE 3:**
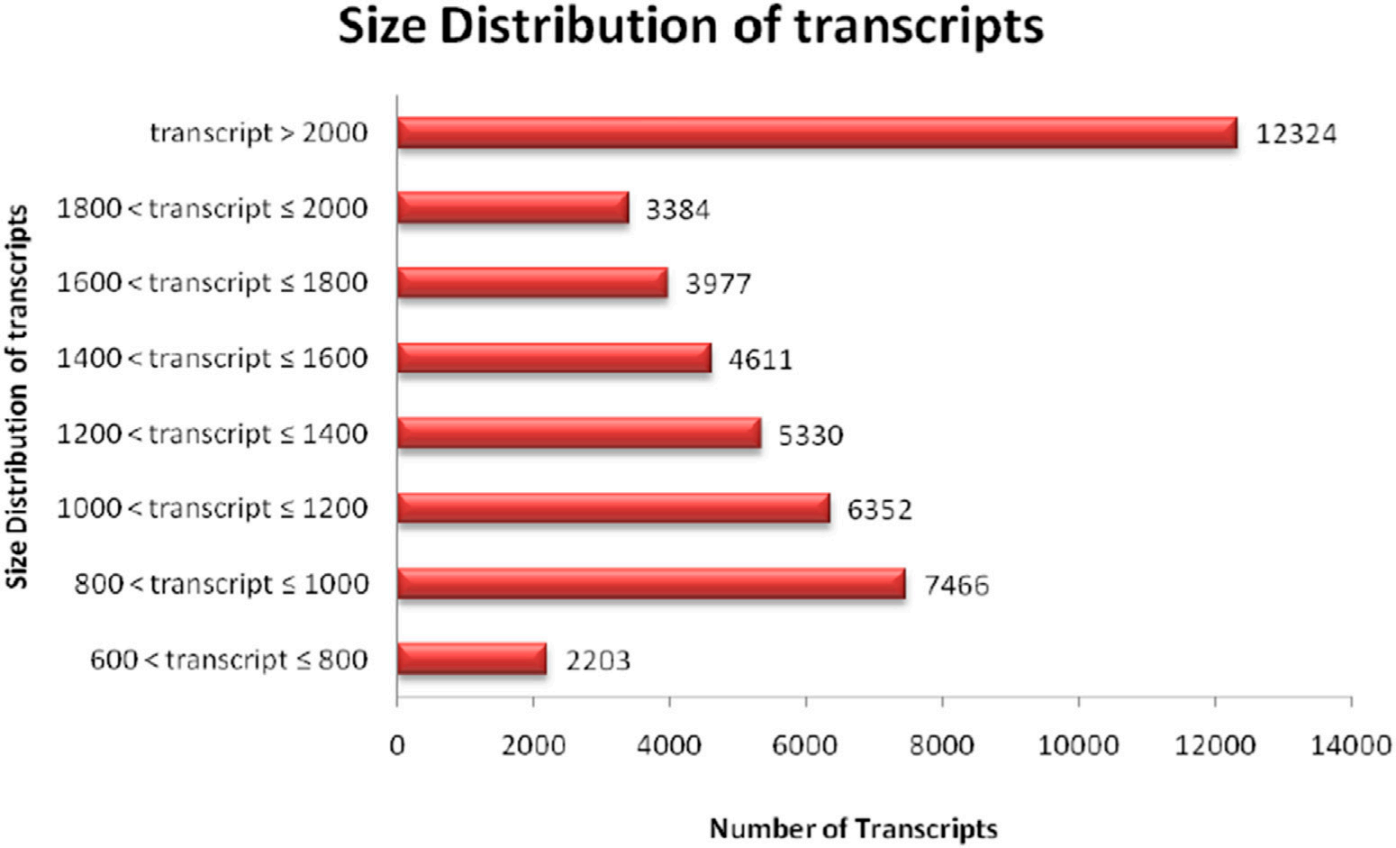
Distribution analysis of transcript length obtained in RNA sequence of NGS-Asparagus sample.

The quality of total RNA was checked on agarose gel-electrophoresis and utilized for *de novo* transcriptome Illumina sequencing analysis (Supplementary Figure S1). Illumina Hi-Seq Platform sequencing results of cDNAs prepared from total RNA resulted in 23,101,000 high-quality reads (2 × 150 bp; 6,667,619,278 nucleotides) with paired-end raw reads (∼6.6 GB) from the pooled assembled transcript of roots and leaf samples of *A. racemosus* (Table 2). The pooled assembly from the two libraries generated 45,647 assembled transcripts comprising 76,398,686bp total transcriptome length with a mean transcript length of 1,674 and N50 of 1,868bp (Table 2). CDS prediction analysis from 45,647 pooled assembled transcripts resulted in 45,444 CDS with a total length and mean length of 76,398,686 and 1,674 bp, respectively (Table 2). Transcripts distribution analysis revealed the highest number of transcripts (12,324) above 2000bp length, followed by 7,466 transcripts in the length range of 800-1,000bp (Table 2; Figure 3). Low numbers of transcripts (2203) were observed with a 600-800bp length range of transcripts. Moreover, the highest number of CDS (11,656) predicted having a CDS length greater than 1200bp, followed by CDS (8979), which had a length distribution in the range of 400-600 bp.

**TABLE 2:** Statistical analysis of whole transcriptome data of *A. racemosus* under methyl jasmonate stress treatment.

Parameters	Asparagus pooled sampled
High-quality reads	23,101,000
Total Number of bases	6,667,619,278
Total data (in Gb)	6.6
Pooled assembly statistics	
Transcript description	**Transcript stat**
Total transcripts	45,647
Total transcript length (bp)	76,398,686
N50	1,868
Maximum transcript length (bp)	7,847
Mean Transcript Length (bp)	1,674
**Length range of transcript**	**No. of transcript**
600 < transcript ≤800	2203
800 < transcript ≤1,000	7,466
1,000 < transcript ≤1,200	6352
1,200 < transcript ≤1,400	5330
1,400 < transcript ≤1,600	4611
1,600 < transcript ≤1800	3977
1800 < transcript ≤2000	3384
Transcript >2000	12,324
**Length of CDS**	**No. of CDS**
200 < CDS ≤400	6421
400 < CDS ≤600	8979
600 < CDS ≤800	7,421
800 < CDS ≤1,000	6272
1,000 < CDS ≤1,200	4695
>1,200	11,656
**The CDS pooled Data Distribution Statistics**	
**Sample Name**	**Pooled CDS**
Total no. of CDS	45,444
# CDS with Blast Hits	42,396
# CDS without Blast Hits	3,048
Domains	No.CDS
Biological process	19,550
Molecular function	19,873
Cellular Component	14,577

### 3.2 Functional annotation and GO classification distribution

**FIGURE 4:**
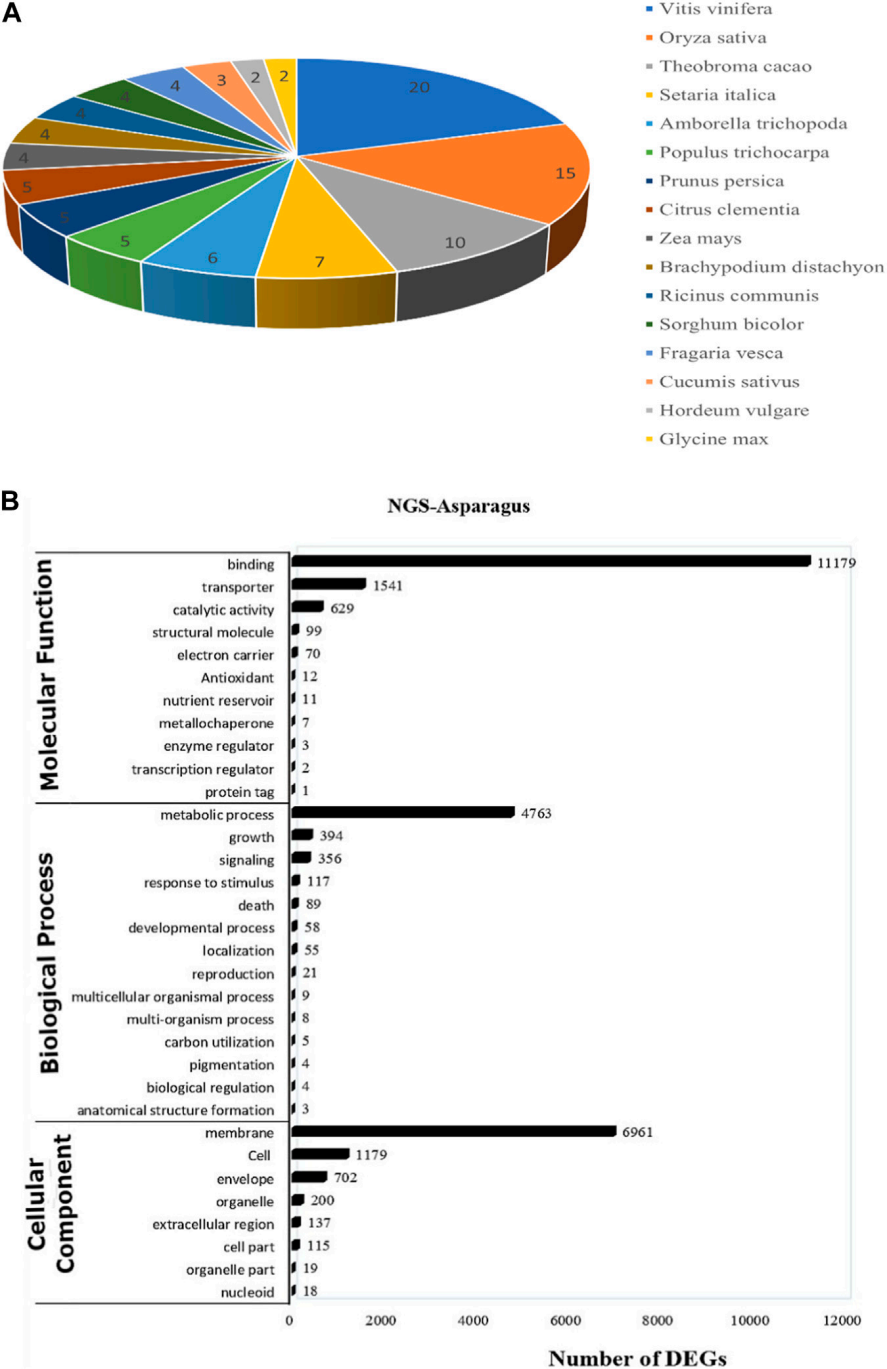
**(A)** The top blast hit species distribution of NGS-Asparagus sample Figure 4B **(B)** Gene ontology functional enrichment analysis across the three domains (Molecular Function, Biological process and Cellular Function) from RNA seq. data of *A. racemosus* under methyl jasmonate stress treatment.

For functional gene analysis, the identified CDS were searched against the NCBI non-redundant (NR) protein database, resulting in the annotation of 42,396 CDS. Among the 45,444 CDS identified, a total of 3,048 CDS were categorized as novel, indicating that they were detected in the RNA-Seq data without any significant hits (Table 2). The majority of the hits were found to be against *Vitis vinifera* (20%), followed by *Oryza sativa* (15%), *Theobroma cacao* (10%), and *Setaria italica* (7%) (Figure 4A). GO mapping was carried out to assign the function for BLASTX annotated CDS using the Blast2GO program. Accordingly, 19,550 CDS were found to be involved in biological processes, 19,873 in molecular function, and 14,577 in cellular components (Table 2; Figure 4). The highest number of CDS (>90%) were involved in binding (11,179) (in the molecular function), metabolic process (4763 CDS) (in biological process) and membrane (6961 CDS) (in the cellular component) (Figure 4B). Some other genes were also identified, including transporter (1541 CDS) and signaling (356 CDS) were involved in biological process and cell (1179 CDS) and envelope (702 CDS) involved in cellular component. Moreover, in molecular function category, the GO distribution assigned CDS related to enzyme encoding involved in the flavonoid’s biosynthetic pathways such as *phenylalanine ammonia-lyase* (13) and *chalcone isomeras*e (6).

### 3.3 Transcription factor analysis

Transcription factors (TFs) are proteins that bind to DNA and control gene expression by activating or inhibiting transcription. Transcription factor analysis generated 80 different transcription factor families in the NGS-Asparagus sample. Transcription factor analysis obtained the highest CDS (905) assigned to Cysteine3Histidine (C3H), followed by a far-red-impaired response (FAR1) (638 CDS) and NAC transcription factors (612 CDS) and bHLH (567) (Figure 7). The NAC family of plant transcription factors is well-known for its involvement in plant stress responses. Additionally, the RNA-Seq data analysis revealed the presence of 454 CDS related to MYB-related transcription factors, which play crucial roles in plant stress responses as well as other biological processes such as development, differentiation, and defense metabolism (Choudhri et al., 2018). Other transcription factors identified were mitochondrial transcription termination factor (mTERF) (206), Tumor necrosis factor receptor-associated factors (TRAFS) 243 (222), Auxin/indole-3-acetic acid (Aux/IAA) (230), MADS (524), C2H2 (408), WRKY (269), SNF2 (294), AP2-EREBP (262), RWP-RK (239), HB (235), FHA (230), *etc.*

### 3.4 KEGG pathway mapping of CDS

The identified CDS were mapped to the reference canonical pathways in KEGG to analyze the involvements of predicted CDS in a particular pathway. Accordingly, the KEGG pathway mapping analysis revealed that 6,353 CDS were enriched in 25 different KEGG pathway categories. A large number of CDS (3,065) were mapped into metabolic pathways, the majority of which were shown to be related to carbohydrate metabolism (524), followed by carbon metabolism (382), amino acid metabolism (381), energy metabolism (340) and lipid metabolism (302) (Table 4). A total of 1,561 CDSs were functionally assigned to genetic information processing, among which 645 CDS were involved in translation, followed by folding, sorting, and degradation (522 CDS) and transcription (394 CDS). Moreover, the KEGG pathway mapping functionally assigned a total of 818 CDS involved in environmental information processing with the highest number (532 CDS involved in signal transduction), 770 CDS assigned to cellular processing with the highest number (323 CDS involved in transport and catabolism), and organismal system (139 CDS involved in environmental adaptation.

#### 3.4.1 Potential CDS identification related to secondary metabolism from KEGG mapping

**FIGURE 5:**
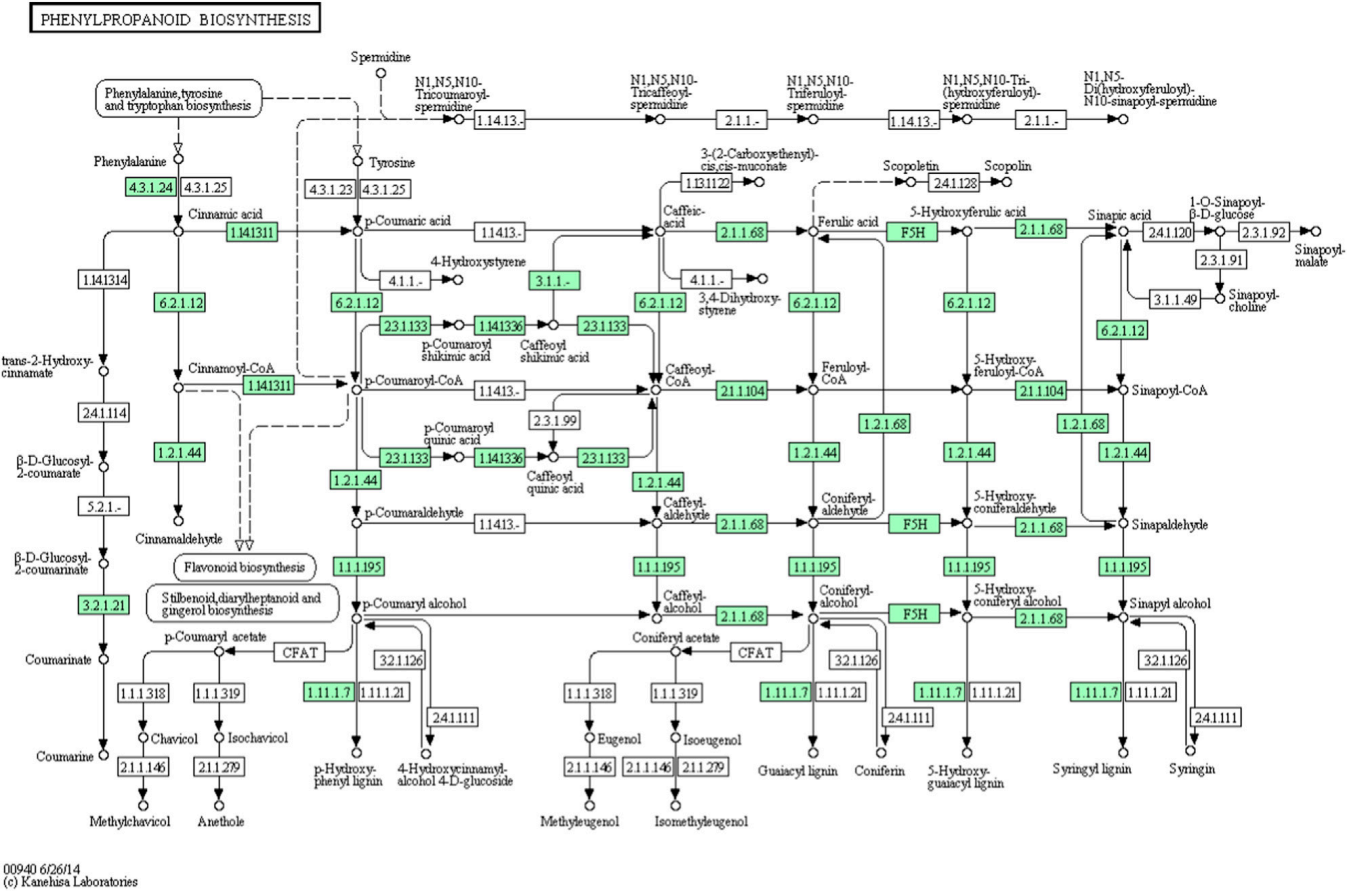
KEGG annotation analysis of metabolic pathway for the phenylpropanoid biosynthesis and involvement of different genes in *A. racemosus* under MeJA treatment.

**FIGURE 6:**
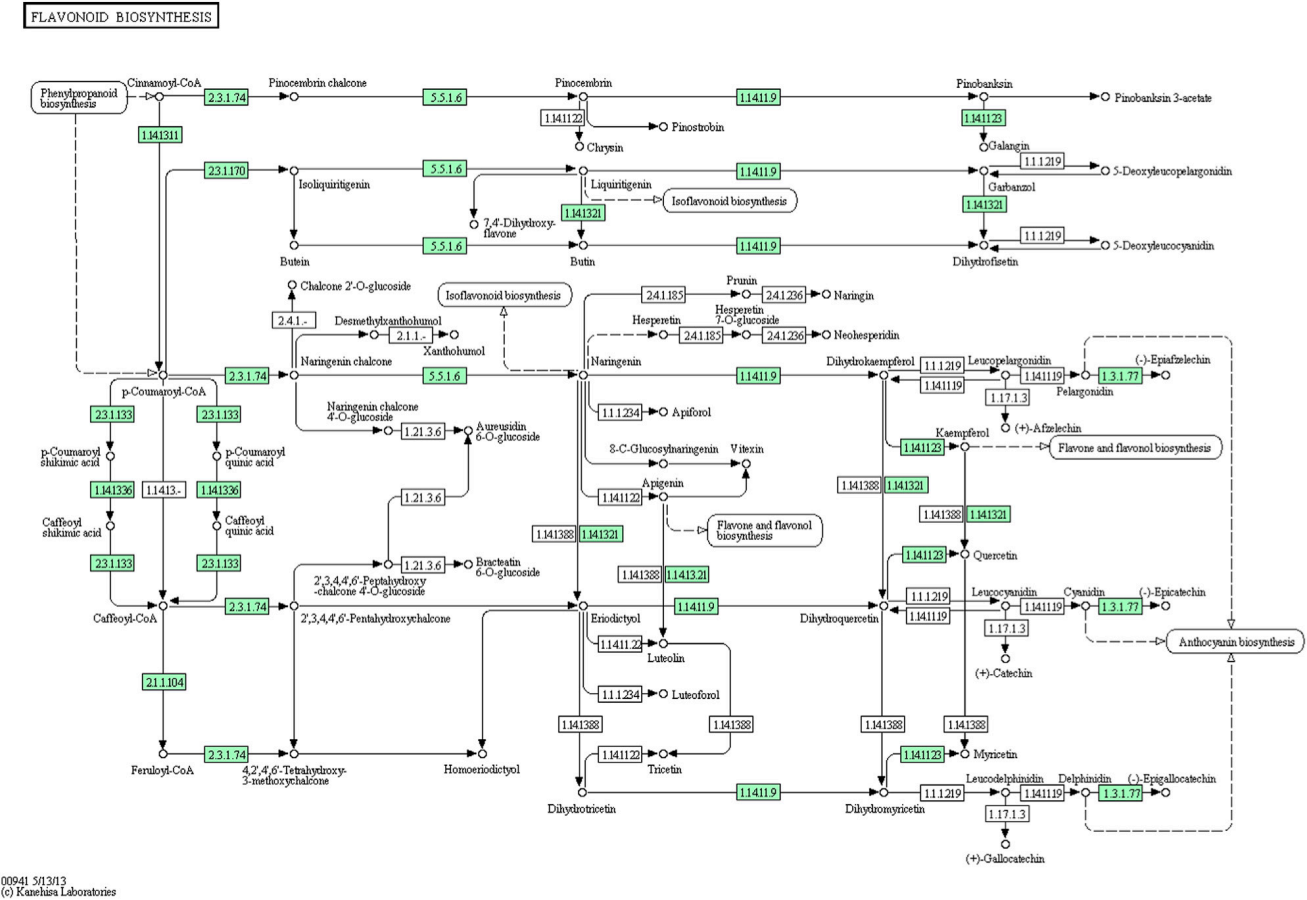
KEGG annotation analysis of metabolic pathway for the biosynthesis of flavonoids and involvement of different genes in *A. racemosus* under MeJA treatment.

From the *A. racemosus de novo* transcriptome sequencing data, a KEGG pathway mapping functionally assigned many CDS involved in the secondary metabolism, i.e., metabolism of terpenoids and polyketides (142 CDS), biosynthesis of other secondary metabolites (117 CDS) and xenobiotics biodegradation and metabolism (65). In the current study, several genes related to phenylpropanoid biosynthesis, flavonoid biosynthesis, and metabolism of terpenoids and polyketides have been identified from *A. racemosus* tissues by KEGG Pathway functional annotation. Among 117 CDSs related to the biosynthesis of other secondary metabolites, CDSs were found to be functionally involved in phenylpropanoid (64), flavonoid biosynthesis pathways (12), anthocyanin biosynthesis (2), isoflavonoid biosynthesis (1), flavone and flavonol biosynthesis (3), respectively (Table 4; Figure 4; Figure 5; Figure 6). An organ and developmentally specific pattern of metabolites is created by a combination of reductases, oxygenases, and transferases, amplifying the resulting hydroxycinnamic acids and esters in several cascades based on the few intermediates of the shikimate pathway as the core unit, generated by the general phenylpropanoid metabolism.

**FIGURE 7:**
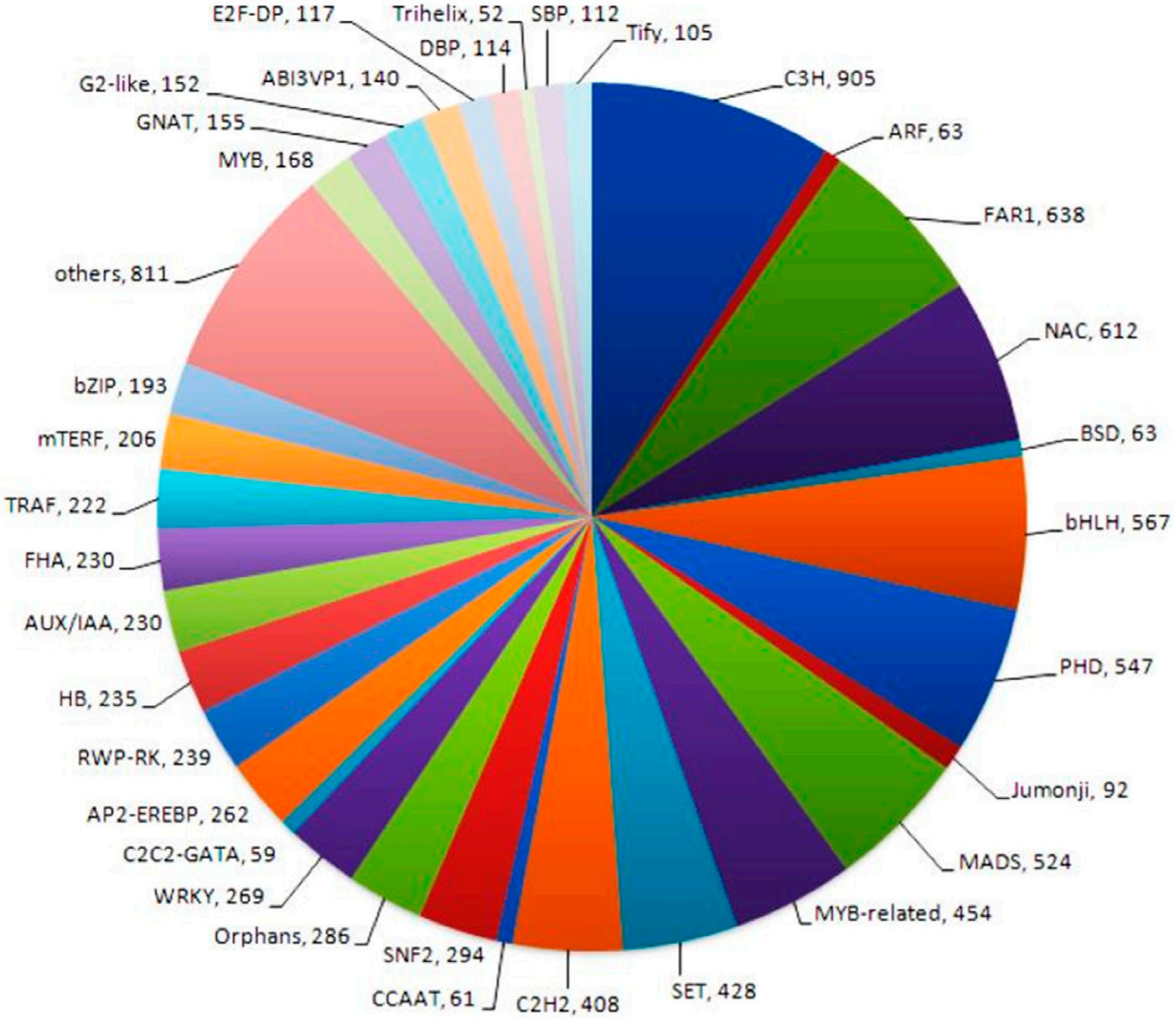
Transcription factor analysis generated 80 different transcription factor families using P2TF NGS-Asparagus sample Fold change.

### 3.5 Mining of SSR marker associated with flavonoid pathway genes

Simple sequence repeats (SSRs) or microsatellites are tandem repeats of nucleotide motifs of sizes one to six bp and are highly polymorphic with a ubiquitous presence in all the known genomes. The simple sequence repeats (SSRs) or micro-satellite associated with flavonoids were picked using MIcroSAtellite Identification Tool (MISA v1.0) from the pooled transcript, resulting in 7,558 SSRs markers. Among 7,558 SSRs markers identified, the highest number of SSR were 3,810, identified as di-nucleotides, followed by trinucleotide with 3,486 SSRs counts (Table 3). Therefore, the di-nucleotide was the most abundant, with a frequency of 50.41%, followed by trinucleotide, with a frequency distribution of 46.12%. The SSR sequences are further filtered and validated by removing terminal SSRs and retaining SSRs with a flanking region of 150bp, resulting in 2917 SSRs predicted to contain a flanking region.

**TABLE 3:** SSR markers analysis from RNA seq data of *A. racemosus* under methyl jasmonate stress treatment.

Description	NGS-Asparagus sample
Total number of Sequence examined	45,647
Total size of examined sequences (bp)	76,398,686
Total number of identified SSRs	7,558
Number of SSRs containing sequences	953
Number of SSRs present in compound formation	452
**Unit Size of SSR**	**Number of SSRs**
Di-nucleotide	3,810
Tri-nucleotide	3,486
Tetra-nucleotide	234
Penta-nucleotide	28
Total	7,558

**TABLE 4:** KEGG pathways classification of CDS from RNA seq. of *A. racemosus* under methyl jasmonate stress treatment.

Pathway category	No. of CDS
**Metabolism**	
Carbon metabolism	382
Carbohydrate metabolism	524
Energy metabolism	340
Lipid metabolism	302
Nucleotide metabolism	241
Amino acid metabolism	381
Metabolism of other amino acids	158
Glycan biosynthesis and metabolism	142
Metabolism of cofactors and vitamins	271
Metabolism of terpenoids and polyketides	142
Biosynthesis of other secondary metabolites	117
Xenobiotics biodegradation and metabolism	65
**Genetic information processing**	
Transcription	394
Translation	645
Folding, sorting and degradation	522
**Environmental information processing**	
Replication and Repair	260
Membrane transport	22
Signal transduction	532
Signaling molecules and interaction	4
**Cellular process**	
Transport and catabolism	323
Cell motility	71
Cell growth and death	257
Cell communication	107
Sensory system	12
**Organismal system**	
Environmental adaptation	139

### 3.6 qRT-PCR analysis of flavonoid biosynthesis pathway-related genes

**FIGURE 8:**
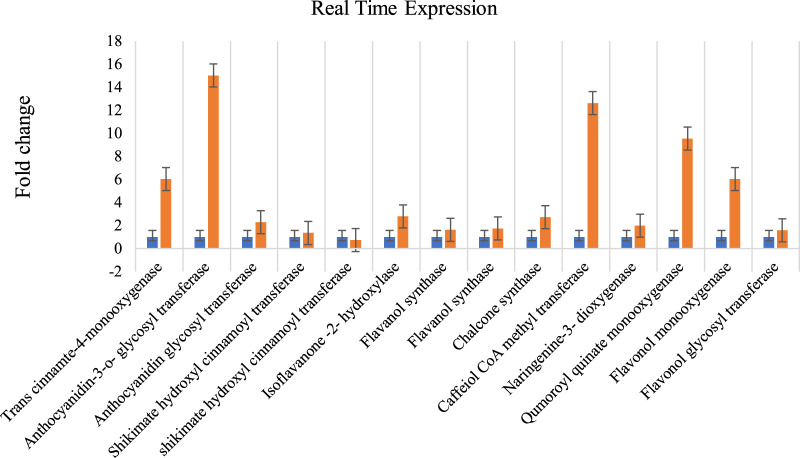
Methyl Jasmonate induced expression of genes involved in flavonoid biosynthesis pathways in *A. racemosus*. Orange colors represent the expression of genes associated with flavonoid biosynthesis pathways in MeJA-treated pooledleaf and root, while blue represents the control plant. Real Time Expression.

The total RNA extracted with three replications each from *A. racemosus* leaf and MeJA-treated leaf samples was observed as 28S and 18S bands on 1% denaturing agarose gel (Supplementary Figure S2). The Nano-drop spectrophotometer analysis resulted in 1,261 ng/μL (1), 1,358 ng/μL (2), 1,193 ng/μL (3), 1,497 ng/μL (4), 1948 ng/μL (5) and 1,584 ng/μL (6) concentrations, respectively. Fourteen genes involved in flavonoid biosynthesis pathways were analyzed for their differential expression from pooled (root and leaf) and methyl jasmonate-treated leaf samples. The qRT-PCR analysis revealed a high-fold expression obtained in the gene encoding anthocyanidin-3-o-glycosyl transferase (FC = 15.029), followed by caffeiol CoA methyltransferase (FC = 12.63) and qumoroyl quinate monooxygenase (FC = 9.55) (Figure 8). Comparatively, a high level of differential gene expression was observed in MeJA-induced expression than in the non-treated leaf sample. A similar expression pattern was observed across all genes involved in the flavonoid biosynthesis pathways in the non-treated leaf samples.

## 4 Discussion

*Asparagus racemosus* is a Rasayana medicine that has been used since the ancient period to treat different diseases. In India, the tuberous root of *A. racemosus* has been traditionally used for medicinal purposes to treat various health conditions. The major secondary metabolites in the roots of *A. racemosus are* flavonoids, alkaloids, and saponins, which attributes to their pharmacological activities. Because of flavonoids, the plant’s roots exhibit anticancer, antioxidant, anti-inflammatory, and anti-viral activities. Despite its applications, the genes involved in flavonoid biosynthesis pathways in *A. racemosus* are largely uncharacterized. Therefore, *de novo* transcriptome sequencing and assembly were performed in the present study to explore genes involved in flavonoid biosynthesis pathways.

A large number of high-quality reads, transcripts, and CDS were obtained from *de novo* transcriptome sequencing data of *A. racemosus*. Accordingly, the current finding obtained ∼2.31 million high-quality reads by paired-end (PE) 2 × 150bp library on Illumina NextSeq in pooled leaf and root libraries, significantly lower than the 60.77 million clean reads reported in *pear calli* (Premathilake et al., 2020). According to Fullwood et al. (2009), to improve the efficacy of next-generation sequencing (NGS) for genome function and variation analysis, paired-end sequencing is significantly beneficial because it increases read length, improves accuracy, and reduces ambiguity compared to single-tag sequencing.

The identified CDS were searched against the NCBI non-redundant (NR) protein database, resulting in the annotation of 42,396 CDS. Most blast hits were found to be against *V. vinifera* and *O. sativa* showing closer relation with *A. racemosus*. Conesa et al. (2005) highlighted utilizing the Blast2GO program in conducting GO mapping, which assigns functions for BLASTX annotated CDS. Blast2GO is a powerful tool that enables automatic and high throughput functional annotation, making it suitable for non-model species genome research. The process of GO mapping includes searching for gene names or symbols in the gene-product tables of the GO database using BLASTX result accession IDs.

Additionally, accession IDs are used to retrieve UniProt IDs from Protein Information Resources (PIR) databases such as RefSeq, SwissProt, GenPept, TrEMBL, PSD, and PDB. Finally, the DBXREF table of the GO database is searched to obtain information on the accession IDs. The current finding functionally assigned many CDS to 25,342 GO terms, i.e., among the three main domains, a high number of CDS were allocated to Molecular Function (19,873), followed by Biological Process (19,550) and Cellular Component (14,577). The present report mapped a small number of CDS involved in molecular function than 99,868 CDS assigned for molecular function reported by Upadhyay et al. (2014) during *de novo* transcriptome analysis of novel genes involved in steroidal sapogenin biosynthesis of *A. racemosus*. In the GO functional annotation, the present finding assigned many GO terms (25,342) to CDS involved in flavonoids biosynthesis pathway, as compared to 7728 GO terms reported involved saponin biosynthesis pathways in the roots, leaves, and fruits of *A. racemosus* (Srivastava et al., 2018), suggests that various types GO terms are involved the flavonoid biosynthesis pathway. Also, in a study conducted by Loke et al. (2017) “Phenylpropanoid biosynthesis” pathway was found to be particularly abundant in the root transcriptome. The process splits off into several significant pathways, including the flavonoid biosynthetic pathway, which contributes to plant resistance (Treutter, 2006). The direct involvement of phenylpropanoids in plant stress responses to temperature, drought, UV radiation, and nutrient deficiency is well established (Korkina, 2007).

KEGG differs from artificial intelligence and machine learning methods as it utilizes the cognitive ability of humans to construct “models” of biological systems, particularly in the form of KEGG pathway maps. These maps are created manually by extracting knowledge from published literature. They can be utilized in extensive biological data analysis to reveal the systemic functions of an organism that may be concealed in its genome sequence. This can be achieved through the straightforward procedure of KEGG mapping (Kanehisa et al., 2022). In a study conducted by Upadhyay et al. (2014), only 162 and 156 unigenes (leaf and root, respectively) were assigned to secondary metabolite pathways. A total of 413 CDS were involved in secondary metabolite synthesis, out of which 82 CDS were involved in different pathways for different flavonoids. A quite high number of unigenes (156) involved in flavonoid synthesis were identified in safflower (Li et al., 2012) and this observed disparity may be attributed to variations in the plant species selected for transcriptome sequencing.

According to Mazid et al. (2011), plants synthesize secondary metabolites to protect themselves from diverse biotic and abiotic stresses. The biosynthesis of these molecules can be induced using chemical elicitors, such as methyl jasmonate, which activate the biosynthetic genes through a complex signaling cascade. Shabani et al. (2009) highlighted that this process occurs at the transcriptional level, whereby methyl jasmonate transcriptionally activates the biosynthetic genes. The genes associated with flavonoid biosynthesis pathways were annotated and validated using qRT-PCR. A total of fourteen genes involved in flavonoid biosynthesis-associated were experimentally validated for their differential expression in pooled leaf and root samples of *A. racemosus* using qRT-PCR. Most of the genes showed differential expressions in MeJA treated leaf sample than in the pooled leaf and root samples, suggesting flavonoid biosynthesis pathway genes are induced in response to the treatment of MeJA. The MeJA induced the expression of genes associated with the flavonoid biosynthesis pathway. The qRT-PCR revealed the differential accumulation genes in response to MeJA. The qRT-PCR resulted in a high-fold expression in the gene encoding an anthocyanidin-3-o-glycosyl transferase (FC = 15.029), followed by caffeiol CoA methyltransferase (FC = 12.63) and qumoroyl quinate monooxygenase (FC = 9.55) compared to the untreated plant. Anthocyanidin-3-o-glycosyl transferase (3 GT) is an enzyme that plays a critical role in the final step of anthocyanin biosynthesis by transferring a glucose, galactose, or arabinose molecule to the hydroxyl group of the anthocyanidin molecule, which enhances the stability and solubility of the anthocyanins (Rainha et al., 2020). Caffeiol CoA methyltransferase (CCoAOMT) catalyzes the transfer of a methyl group from S-adenosyl-L-methionine (SAM) to the hydroxyl group of a phenolic compound, resulting in the formation of a methylated derivative. In flavonoid biosynthesis, CCoAOMT is involved in the conversion of caffeoyl-CoA to feruloyl-CoA, which is a key intermediate in the biosynthesis of a range of flavonoids (Hegde et al., 2021). Qumoroyl quinate monooxygenase (CYP98A14) is a cytochrome P450 monooxygenase that catalyzes the hydroxylation of quinate to produce caffeoylquinate, a precursor of chlorogenic acid and other phenolic compounds. In flavonoid biosynthesis, CYP98A14 is involved in the hydroxylation of p-coumaroylshikimate to form caffeoylshikimate, which is a key intermediate in the biosynthesis of a range of flavonoids, including anthocyanins, flavonols, and proanthocyanidins (Yang et al., 2022). This indicated that these three genes have significantly impacted the flavonoid biosynthesis pathway.

The qRT-PCR result exhibited low accumulations of genes encoding flavonol glycosyl transferase, flavanol synthase, and shikimate hydroxyl cinnamoyl transferase (cds_14257) in the MeJA-treated leaf sample. However, the gene encoding shikimate hydroxyl cinnamoyl transferase (cds_14258) was found to be downregulated in the MeJA-treated leaf sample. Upadhyay et al. (2014) found that the application of methyl jasmonate leads to an increase in plant accumulation by inducing gene transcripts related to secondary metabolites. Similarly, Premathilake et al. (2020) reported the upregulation of flavonoid biosynthesis pathway structural genes (PcCHS, PcCHI, PcF3H, PcDFR, PcANS, PcANR2a, and PcLAR1) following treatment with MeJA. Du et al. (2021) studied the effect of methyl jasmonate on genes involved in flavonoid biosynthesis in pigeon pea plant and it was found that various genes including Caffeiol CoA methyltransferase is induced by methyl jasmonate that supports current findings.

MISA microsatellite finder is a software application that is utilized for identifying microsatellites present in nucleotide sequences. It is not just limited to detecting flawless microsatellites, but is also able to identify perfect compound microsatellites, which comprise multiple instances of more than one simple sequence motif (Beier et al., 2017). In the present study, MIcroSAtellite Identification Tool (MISA v1.0) from the pooled transcript resulted in 7,558 SSRs markers in the NGS-Asparagus sample, lower than the identified 18,107 in the leaf and 26,733 SSRs in the root of *A. racemosus* involved in steroidal sapogenin biosynthesis in *A. racemosus* (Upadhyay et al., 2014). Furthermore, the di-nucleotide was exhibited as the most abundant, with a frequency of 50.41%, followed by trinucleotide, with a frequency distribution of 46.12%, which is not in agreement with the report of Upadhyay et al. (2014), which tri-nucleotide repeats were the most abundant SSR motif in leaf tissues followed by di-nucleotide. However, Srivastava et al. (2018) reported that mononucleotide (p1) SSR (50.93%, 20,800) was abundantly identified, followed by di-nucleotide (p2) (22.91%, 9356) and trinucleotide (p3) (17.37%, 7,096).

The transcriptome of *A. racemosus* was examined to identify transcripts encoding transcription factors (TFs). These TFs are part of various multi-gene families. They are essential in regulating the expression of individual or multiple genes by binding to specific sequences in the promoter regions of target genes, known as cis-acting elements*.* (Broun et al., 2006; Pérez-Rodríguez et al., 2010). The current study revealed that the highest CDS (9.03%, 905) was assigned to Cysteine-3- Histidine (C3H), followed by a far-red-impaired response (FAR1) (6.37%, 638 CDS) and NAC transcription factors (6.11%, 612 CDS). The transcription factors belonging to the C3H-type zinc finger family are crucial in various aspects of plant growth and development and in their ability to adapt to biotic and abiotic stressors (Liu et al., 2020). FAR1 and NAC transcription factors families are more expressive in plants during stress conditions (Tran et al., 2007; Hou et al., 2023). However, Srivastava et al. (2018) reported the most significant members of transcription factors TIG (1,241, 10.55%), followed by FAR1 (925, 7.86%), C3H (785, 6.67%), MYB/MYB related (680, 5.78%). The present study assigned 6.37%, 638 CDS to C3H and 4.54%, 454 CDS, which is associated with Srivastava et al. (2018) reports that C3H and MYB/MYB related assigned (785, 6.67%) and (680, 5.78%) in *A. racemosus* transcriptome, respectively. The transcriptome analysis of *A. racemosus* further revealed the presence of several transcription factors (TF) families, including AP2-EREBP, bHLH, C2C2, MYB/MYB related, and WRKY, which have been previously reported to regulate the biosynthesis of secondary metabolites in plants (Kato et al., 2007; Suttipanta et al., 2011; Zhang et al., 2011). The present finding could give an insight into the molecular understanding of the gene involved in the flavonoid biosynthesis pathway and helps for further secondary metabolic improvement in the *A. racemosus*.

## 5 Conclusion

The EST data obtained from *de novo* transcriptome sequencing provides the foundation for functional genomics research in *A. racemosus*. The annotated transcripts provide valuable information for studying the biochemical, cellular, and molecular aspects of the *A. racemosus* transcriptome. This knowledge can aid in metabolic engineering and elucidate the regulation of secondary metabolic biosynthetic pathways in plants. The gene encoding anthocyanidin-3-o-glycosyl transferase, caffeiol CoA methyltransferase, and qumoroyl quinate monooxygenase have a significant role in the biosynthesis of the flavonoid pathway. Methyl jasmonate has been found to be an effective inducer of flavonoid biosynthesis transcripts in plants, resulting in the upregulation of genes encoding enzymes involved in flavonoid biosynthesis. The expression of the gene encoding Shikimate hydroxyl cinnamoyl transferase (cds_14258) was inhibited by the treatment of MeJA and downregulated in the treated sample. Understanding the regulation of flavonoid biosynthesis and the key enzymes involved in this process can aid in developing new strategies for plant metabolic engineering and producing high-value flavonoid compounds. The limitation of current study is that it only provides information on a part of the whole picture as only a small number of potential genes involved in flavonoid biosynthesis were chosen for research. Although there are various other candidate genes which are involved in flavonoid biosynthesis identified by *de novo* sequencing and are worthy of further investigation. Consequently, it makes it hard to draw precise conclusions. However, the dataset will provide an important resource foundation for future genetic or genomes studies on *Asparagus* species and will help to give better insight into the mechanism of biosynthesis of flavonoids in *A. racemosus*. This information can significantly help genetically improve and identify germplasm with high alkaloids, flavonoids and saponins.

## Data Availability

The original contributions presented in the study are publicly available. This data can be found here: https://www.ncbi.nlm.nih.gov/bioproject/. Accession number: PRJNA977204.
