# Supplementary material for: De novo sequencing, assembly, and characterization of Asparagus racemosus transcriptome and analysis of expression profile of genes involved in the flavonoid biosynthesis pathway
Source: Front Genet. 2023 Sep 7;14:1236517. doi: 10.3389/fgene.2023.1236517 (PMC10513371; doi:10.3389/fgene.2023.1236517)
Supplement: Supplementary file 1 [file Image1.pdf]

De novo sequencing, assembly, and characterization of _Asparagus racemosus_ transcriptome and analysis of expression profile genes involved in the flavonoid biosynthesis pathway

Supplementary Figures

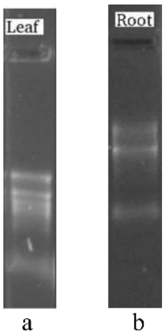

Supplementary Fig. 1. Quality of total RNA was checked on 1% denatured agarose gel; (a) Quality of total RNA isolated from leaf; (b) Quality of total RNA isolated from root.

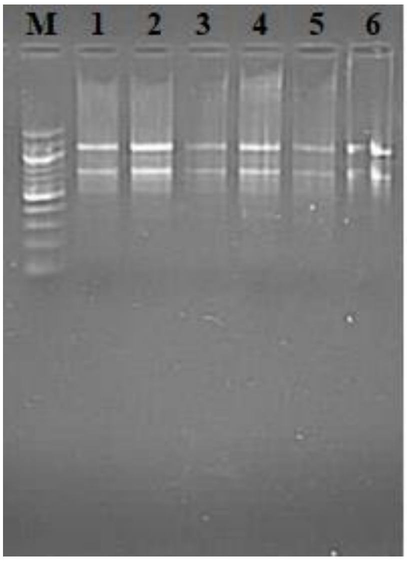

Supplementary Fig. 2. Quality of total RNA isolated with three replications each from *A. racemosus* leaf (lane 1,2,3) and MeJA-treated leaf samples (lane 4,5,6) in 1% denatured agarose gel. M is 100bp DNA ladder.
